# RocA Truncation Underpins Hyper-Encapsulation, Carriage Longevity and Transmissibility of Serotype M18 Group A Streptococci

**DOI:** 10.1371/journal.ppat.1003842

**Published:** 2013-12-19

**Authors:** Nicola N. Lynskey, David Goulding, Magdalena Gierula, Claire E. Turner, Gordon Dougan, Robert J. Edwards, Shiranee Sriskandan

**Affiliations:** 1 Faculty of Medicine, Imperial College London, Hammersmith Hospital, London, United Kingdom; 2 Wellcome Trust Sanger Institute, Wellcome Trust Genome Campus, Hinxton, Cambridge, United Kingdom; National Institute of Allergy and Infectious Diseases, National Institutes of Health, United States of America

## Abstract

Group A streptococcal isolates of serotype M18 are historically associated with epidemic waves of pharyngitis and the non-suppurative immune sequela rheumatic fever. The serotype is defined by a unique, highly encapsulated phenotype, yet the molecular basis for this unusual colony morphology is unknown. Here we identify a truncation in the regulatory protein RocA, unique to and conserved within our serotype M18 GAS collection, and demonstrate that it underlies the characteristic M18 capsule phenotype. Reciprocal allelic exchange mutagenesis of *rocA* between M18 GAS and M89 GAS demonstrated that truncation of RocA was both necessary and sufficient for hyper-encapsulation via up-regulation of both precursors required for hyaluronic acid synthesis. Although RocA was shown to positively enhance *covR* transcription, quantitative proteomics revealed RocA to be a metabolic regulator with activity beyond the CovR/S regulon. M18 GAS demonstrated a uniquely protuberant chain formation following culture on agar that was dependent on excess capsule and the RocA mutation. Correction of the M18 *rocA* mutation reduced GAS survival in human blood, and *in vivo* naso-pharyngeal carriage longevity in a murine model, with an associated drop in bacterial airborne transmission during infection. In summary, a naturally occurring truncation in a regulator explains the encapsulation phenotype, carriage longevity and transmissibility of M18 GAS, highlighting the close interrelation of metabolism, capsule and virulence.

## Author Summary

Group A streptococcus is an important human pathogen which produces a polysaccharide capsule that confers resistance to killing by white blood cells and allows bacterial adherence to host epithelial surfaces. Serotype M18 isolates over-produce capsule, creating a unique and characteristic appearance when grown on blood agar. This feature may underlie the waves of infectious pharyngitis and subsequent onset of rheumatic fever associated with this serotype. The reason for hyper-encapsulation of M18 GAS is unknown. Here we show that a naturally-occurring truncation in an important regulatory protein, RocA, underlies serotype M18 hyper-encapsulation. By correcting the truncation we were able to reverse hyper-encapsulation, modify the 3-D structural morphology of bacteria within colonies and alter the overall protein expression pattern of the bacterium. We were able to reproduce characteristics of M18 streptococci in a different serotype strain by introducing the same truncation mutation. It was also possible to show that the truncation in RocA led to prolonged nasopharyngeal carriage of GAS in mice and also promoted bacterial airborne transmission. Thus, the propensity for M18 isolates to be associated with outbreaks of pharyngitis and rheumatic fever may be accounted for by the level of encapsulation induced by truncation of the regulatory protein RocA.

## Introduction

The group A streptococcal (GAS) hyaluronic acid (HA) capsule is a key virulence determinant that enhances bacterial resistance to neutrophil-mediated opsonophagocytosis and facilitates adherence to epithelial surfaces [1]–[5]. Serotype M18 GAS display a uniformly mucoid, hyper-encapsulated phenotype and have been implicated in outbreaks of pharyngitis and subsequent onset of acute rheumatic fever (ARF) [6]–[10], an immunologically-mediated post-infection sequela to streptococcal tonsillitis that is the leading cause of valvular heart disease globally [11]. To date, the molecular basis for excessive capsule production by M18 GAS strains has remained unknown [12]. Whilst exposure to human blood or animal passage can induce an increase in GAS encapsulation such stimuli do not account for the phenotype exhibited by M18 GAS [2], [13].

HA is comprised of repeating subunits of two hexamers; glucuronic acid and N-acetylglucosomine that are polymerized by HA synthase, the gene for which is located in the HA synthase (*has*) operon [14], [15]. The operon encodes three enzymes required for the production of the HA precursor glucuronic acid; *hasA* (hyaluronate synthase), *hasB* (UDP-glucose dehydrogenase) and *hasC* (a UDP-glucuronic acid uridyl transferase) which are co-transcribed. The second monomer, N-acetylglucosamine is a metabolite produced during cell wall peptidoglycan synthesis [15]. Some isolates of GAS (M4 and M22) lack the *hasABC* operon and therefore do not produce HA capsule indicating that capsule may not be essential for pathogenicity in all serotypes [16]. Whilst single nucleotide polymorphisms at sites in the *has* operon have been demonstrated to impact on the level of GAS encapsulation [12], they do not account for the excessive level of HA produced by strains of serotype M18.

GAS pathogenicity is reliant on transcriptional regulation of virulence factors [17]–[23]. To achieve this, GAS employ a number of two-component regulatory systems (TCS), which together form a highly complex regulatory network. The best studied GAS TCS is the control of virulence operon (CovR/S), also known as CsrR/S, which regulates approximately 10% of the GAS genome [24]. Phosphorylation of cytosolic regulator CovR by its membrane-bound cognate sensor kinase CovS induces enhanced binding to, and repression of, target gene promoters. CovR is an important transcriptional repressor of the capsule synthesis operon (*has* operon) [25]. Whilst its mechanism of action has been rigorously studied, much is unknown regarding the complex interactions between CovR/S and other regulatory proteins. Naturally occurring loss of function mutations in CovR/S induce a hyper-invasive phenotype often associated with enhanced virulence factor expression, including capsule synthesis [26], [27]. However CovR/S mutations do not account for levels of hyper-encapsulation observed in serotype M18 GAS, suggesting an alternative genetic basis for this phenotype. A positive regulator of CovR, RocA, has been reported to up-regulate *covR* transcription with subsequent enhanced repression of capsule synthesis [28]. In this work we identify a unique truncation of RocA in M18 GAS that accounts for the unusual phenotype demonstrated by strains of this serotype in our study.

## Results

### Serotype M18 is phenotypically distinct from other GAS

**Figure 1:**
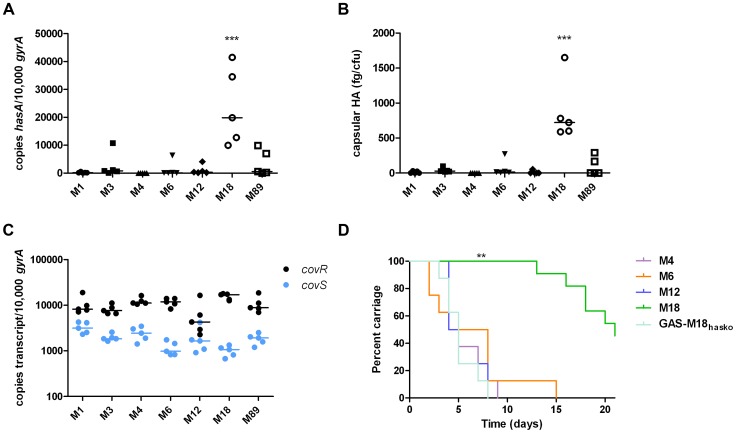
Serotype M18 GAS display a unique hyper-encapsulation phenotype which mediates enhanced nasopharyngeal carriage longevity. Characterization of serotype M18 GAS by comparison with representatives of each of six major serotypes (n = 5 isolates/group). (A) Inter-serotype comparison of absolute copy number for *hasA* standardized to housekeeping gene *gyrA*. Line shows median (Kruskal-Wallis; *** = p<0.001). (B) Inter-serotype comparison of capsular HA expression across all serotypes by an ELISA-based assay. Line shows median (Kruskal-Wallis; *** = p<0.001). (C) Inter-serotype comparison of absolute transcript copy number for *covR* and *covS* standardized to house-keeping gene *gyrA*. Line shows median (Kruskal-Wallis; p = NS). (D) Inter-serotype comparison of duration of nasopharyngeal shedding of pharyngitis-associated serotypes M4, M6, M12 and M18 with isogenic capsule disruptant mutant GAS-M18_hasko_ (n = 8 mice/group). Data represent percentage of mice shedding each strain for 21 days following intra-nasal challenge (LogRank; ** = p<0.01).

Several reports support the observation that M18 GAS strains are more encapsulated than other serotypes [1], [2], [12]. To systematically investigate this, we compared *hasA* transcription and capsular HA synthesis in clinical isolates representing the UK's commonest M-types [M1, 3, 4, 6, 12, 89] with M18 GAS, using five isolates of each (Table 1). M18 GAS strains produced significantly greater amounts of capsular HA than all other types, and this could be attributed to enhanced *hasA* transcription (Figure 1 A and B).

**Table 1:** Clinical isolates used in this study.

Serotype	Strains used
M1	H327^c^ ^r^, H368^c^ ^r^, H506, H739, H742
M3	H325^c^ ^r^, H356, H459, H460, H517
M4	H317, H365, H627, H679^c^ ^r^ #, H897
M6	HM2, HM55, H427, H682^c^ ^r^ #, H693
M12	H357, H529, H530, H599, H600, *H675* c r #
M18	H410^c^ ^r^, H412^c^ ^r^, H563^c^ ^r^, H565^c^ ^r^, H566^c^ ^r^ #, H567^c^ ^r^, *H498* * c r, *H499* * c r, *H414* c r, *H686* r, *H695* r
M89	H293^c^ ^r^, H395^c^ ^r^, H541, H542, H636

All strains were used for *in vitro* capsule quantification and *hasA* and *covRS* transcription assays except those in italics. Strains H566 and H293 are referred to in the text as GAS-M18 and GAS-M89 respectively.

* denotes isolates from 1934;

c denotes strains that underwent *covR/S* sequencing;

r denotes strains that underwent *rocA* sequencing;

# denotes strains used in mouse nasopharyngeal carriage experiments

Given that transcription from the *has* operon is regulated by CovR/S [29], we hypothesized that transcriptional variation in either component could play a role in establishing the M18 phenotype. Overall, however, no difference was observed in inter-serotype transcript levels of either gene when measured by quantitative real time PCR. Despite predictions that *covR* and *covS* are co-transcribed [29], transcript levels of *covR* were, on average, 7-fold higher than *covS* (Figure 1 C) consistent with either differences in gene transcription or differential RNA stability.

The human nasopharynx is a key GAS reservoir and a primary site for both bacterial colonization and persistence. Longevity of naso-pharyngeal carriage was compared between GAS-M18 and other pharyngitis-associated GAS serotypes M4, 6 and 12 in a murine model (Figure 1 D). GAS-M18 colonized the mouse nasopharynx for longer than all other strains tested, with greatest bacterial shedding from the nares (Figure 1 D). To determine whether the HA capsule could account for the observed M18 carriage phenotype, we created an isogenic capsule disruption mutant, GAS-M18_hasko_ (Table 2). Abolition of capsular HA synthesis reduced carriage longevity of GAS-M18 to levels observed for other GAS serotypes tested (Figure 1 D), demonstrating the GAS capsule to be a key mediator of serotype M18 persistence in the nasopharynx.

**Table 2:** Isogenic strains used in this study.

Strain	Plasmid	Gene alteration	Capsule level
GAS-M18	-	Identical to H566	+++
GAS-M18_pcontrol_	pDL278	none	+++
GAS-M18_pcovRM89_	pDL_covRM89_	Over-express CovR_M89_	++
GAS-M18_procAM89_	pDL_rocAM89_	Over-express RocA_M89_	+
GAS-M18_procAM18_	pDL_rocAM18_	Over-express RocA_M18_	+++
GAS-M18_rocAM89_	pUCMUT_rocAM89_AE	RocA_M18_ allelic replacement with RocA_M89_	+
GAS-M18_hasKO_	pUCMUT_hasKO_	Capsule locus disruption mutant	−
GAS-M89	-	Identical to H293	−/+
GAS-M89_rocAM18_	pUCMUT_rocAM18_AE	RocA_M89_ allelic replacement with RocA_M18_	++

### A unique mutation in RocA underlies serotype M18 hyper-encapsulation

**Figure 2:**
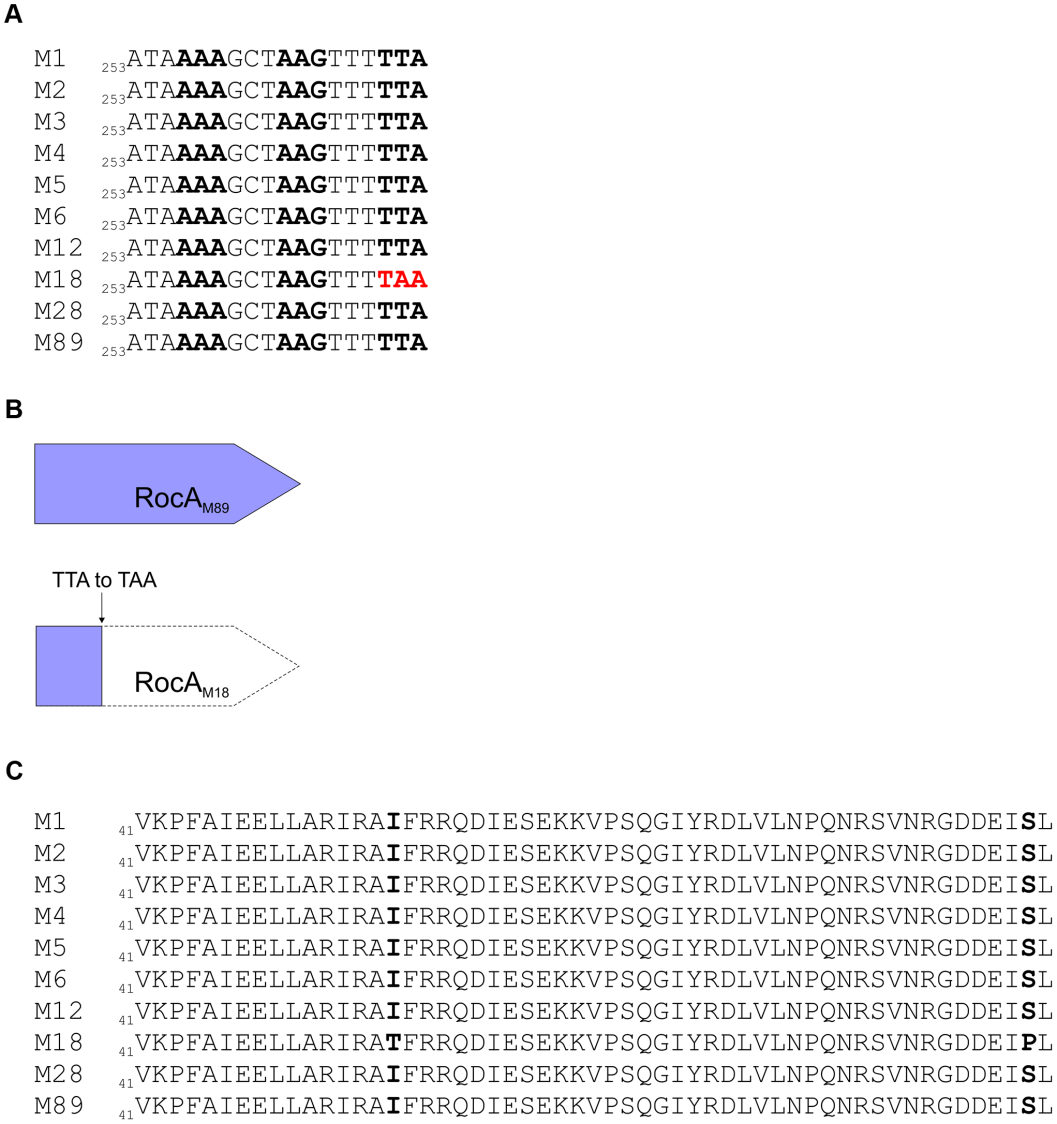
Identification of unique serotype M18 specific mutations in regulators RocA and CovR. RocA and CovR sequence comparison of isolates representing major GAS serotypes. Strains sequenced from each serotype are outlined in Table 1; at least one clinical isolate was tested for each serotype. Ten serotype M18 strains were sequenced including two obtained from patients in 1934. (A) *rocA* gene sequence of different M types. Codons are shown by alternating bold text. The premature stop codon in *rocA*_M18_ is highlighted by red font. (B) The subsequent truncation in RocA_M18_ protein is demonstrated schematically in comparison with RocA_M89_. (C) CovR amino acid sequence in different M types. Highlighted residues indicate amino acid change in the M18 CovR protein resulting from non-synonymous mutations in the gene sequence compared with other M types. RocA and CovR sequences were also evaluated from genome sequenced isolates submitted to NCBI, representing serotypes M1, M2, M3, M4, M5, M6, M12, M18, M28 and M49.

Systematic sequence analysis of a number of known and predicted regulatory GAS genes revealed a serotype M18 specific variation in the nucleic acid sequence for regulator of CovR, *rocA*, with a conserved single nucleotide polymorphism (SNP) from T to A at base pair 269 in the *rocA* nucleotide sequence. This translated into a non-synonymous change from leucine (TTA) to a premature stop codon (TAA) (Figure 2 A) resulting in truncation of the RocA protein at amino acid 90/451 (Figure 2 B). Two non-synonymous mutations were also identified in *covR* (Figure 2 C). Both sets of mutations were unique to and conserved within all M18 isolates analyzed, including contemporary isolates from the UK as well as two pre-antibiotic era UK isolates from 1934 (Table 1), and the USA genome sequenced strain MGAS8232 [7]. The mutations were not identified in the other non-M18 strains tested, nor in genome sequenced strains representing serotypes M1, M2, M3, M4, M5, M6, M12, M28 or M49. To analyze the impact of these previously unexplored mutations on GAS-M18 capsule synthesis, we firstly used plasmids to over-express a full-length functional copy of either *covR* or *rocA* amplified from GAS-M89 or truncated *rocA* amplified from GAS-M18 in GAS-M18, creating strains GAS-M18_pcovRM89_, GAS-M18_procAM89_ and GAS-M18_procAM18_ respectively. As a control GAS-M18 was also transformed with empty plasmid only, yielding GAS-M18_pcontrol_ (Table 2).

**Figure 3:**
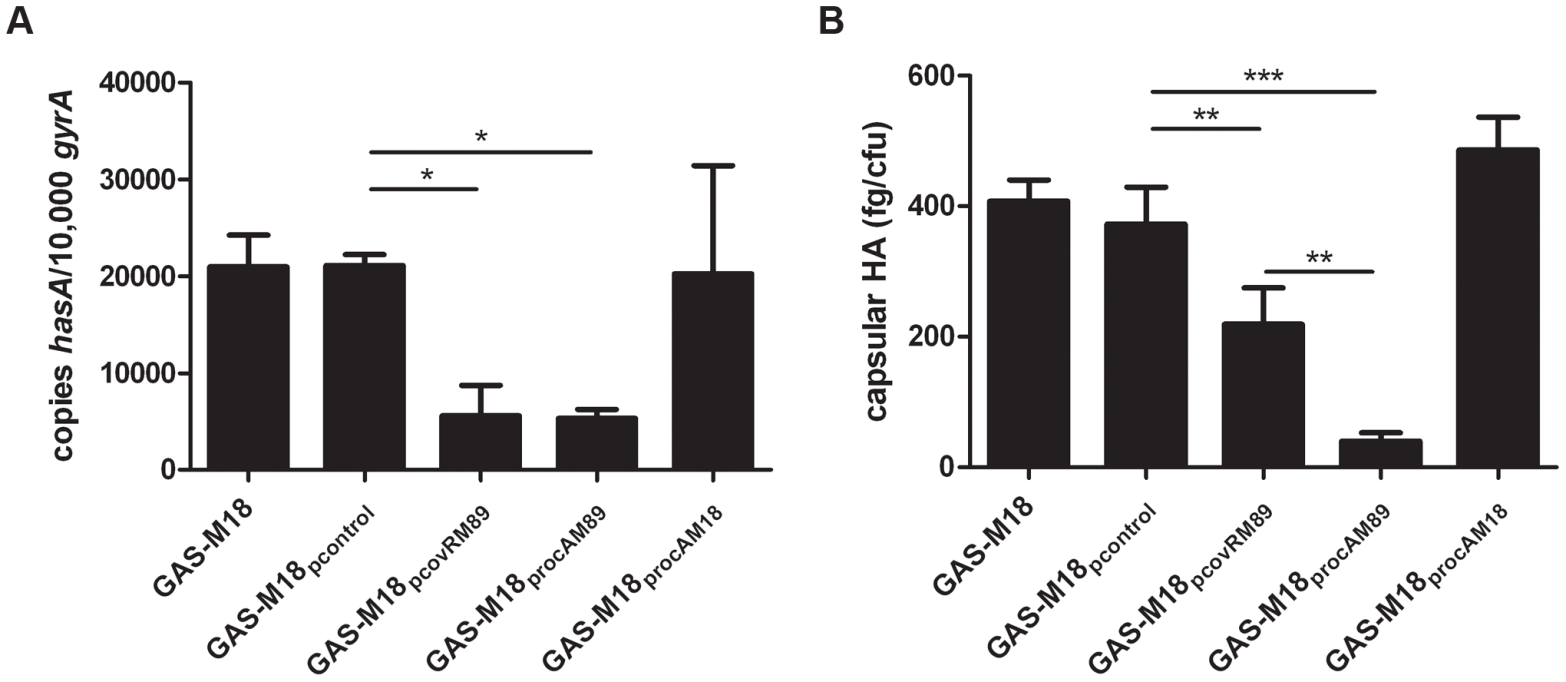
Over-expression of RocA_M89_ but not CovR_M89_ is sufficient to reverse serotype M18 hyper-encapsulation. Analysis of GAS-M18 strains over-expressing *rocA*_M89_, *rocA*_M18_ and *covR*_M89_. (A) Absolute copy number of *hasA* transcripts quantified relative to housekeeping gene *gyrA*. Data shown from mid-logarithmic (ML) growth phase. (B) Capsular HA production was quantified at ML by ELISA. Data represent mean and standard deviation of three independent experiments measured in triplicate (ANOVA with Bonferroni pairwise comparison;* = p<0.05, ** = p<0.01, *** = p<0.001).

Analysis of isogenic strains by quantitative real-time PCR revealed that *hasA* transcription was reduced by over-expression of *covR*_M89_ or *rocA*_M89_ compared with controls (Figure 3 A). This was likely due to enhanced expression and subsequent activity of the CovR repressor, either directly by over-expression of *covR* in GAS-M18_pcovRM89_, or indirectly by RocA mediated up-regulation of *covR* transcription in GAS-M18_procAM89_. However, complete reversal of the M18 hyper-encapsulation phenotype was only seen in GAS-M18_procAM89_, presumably by restoration of HA synthesis regulation (Figure 3 B). This highlighted a major role for RocA in the loss of transcriptional regulation of capsule synthesis in serotype M18 GAS. No difference was observed in either *hasA* transcript levels or capsular associated HA between strains GAS-M18 and GAS-M18_procAM18_ (Figure 3 A and B). The RocA_M18_ truncation was therefore hypothesized to release the *has* operon from CovR repression, however the effects on *hasA* alone were insufficient to fully explain the impact of the RocA truncation on production of capsular HA by GAS-M18.

**Figure 4:**
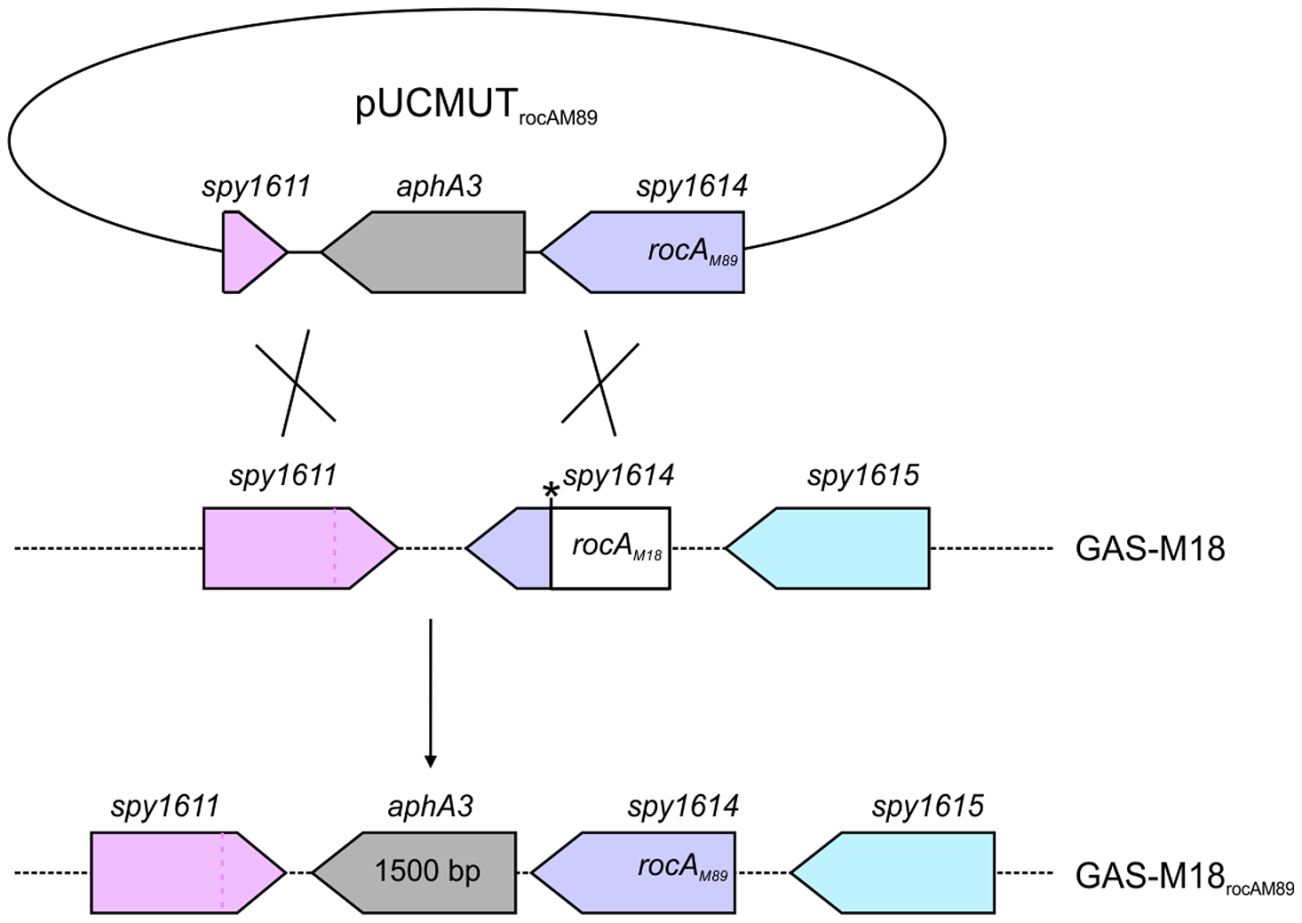
Allelic exchange mutagenesis of *rocA*_M18_ with *rocA*_M89_ in GAS-M18. Correction of the *rocA*_M18_ premature stop codon was achieved by allelic exchange of chromosomal *rocA*_M18_ with *rocA*_M89_ using suicide vector pUCMUT [46]. Double recombination events between *rocA* (*spy1614*) and 500 bp region of downstream gDNA including the 3′ end of *spy1611* resulted in replacement of *rocA*_M18_ with a single copy of *rocA*_M89_, producing isogenic strain GAS-M18_rocAM89_. Allelic exchange was confirmed by PCR and sequencing.

**Figure 5:**
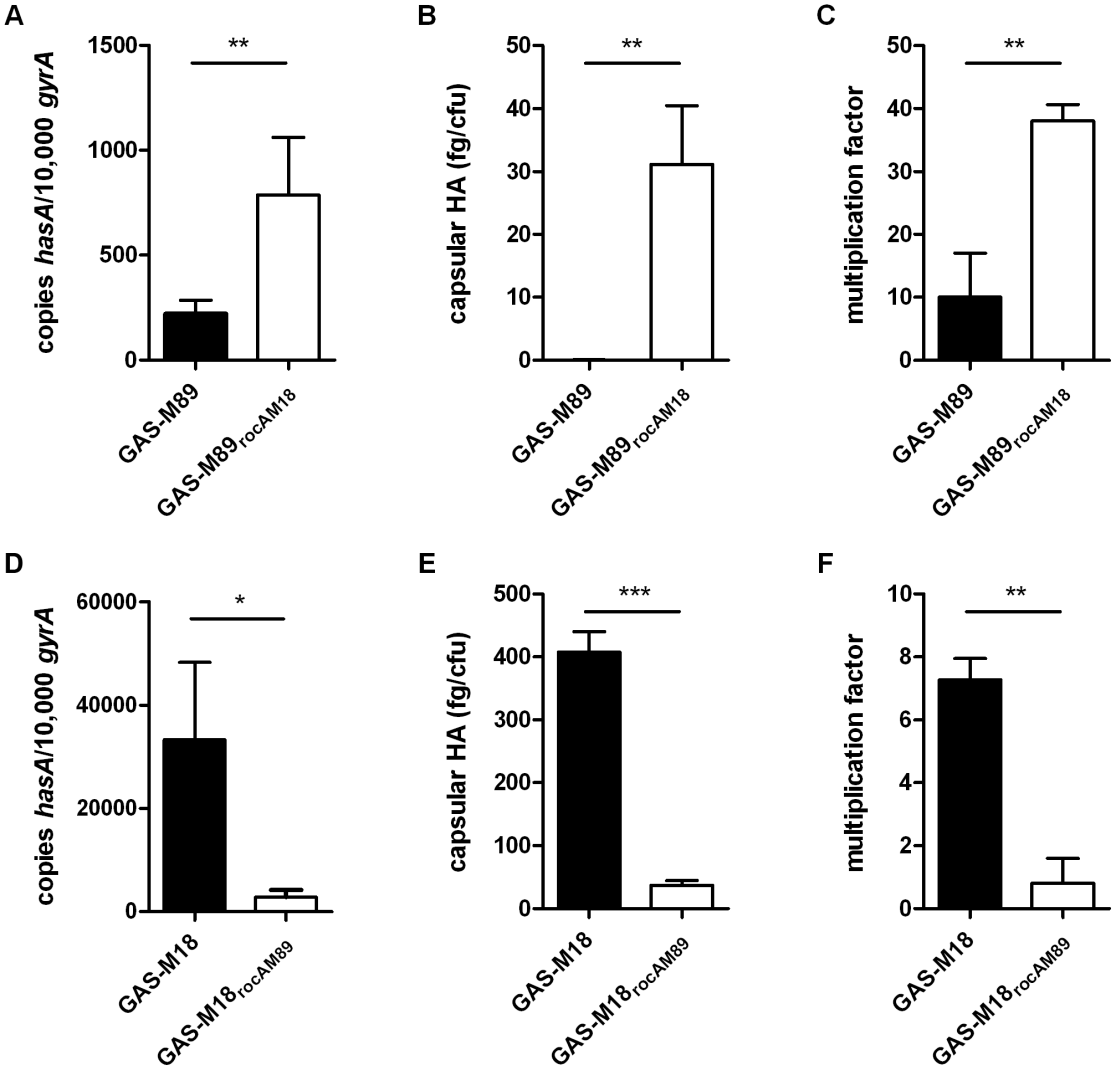
RocA_M18_ truncation is necessary and sufficient for hyper-encapsulation. (A–C) Analysis of the impact of single gene replacement of *rocA*_M89_ with *rocA*_M18_ in strain GAS-M89. (A) Absolute copy number of *hasA* transcripts quantified at early logarithmic (EL) growth phase relative to housekeeping gene *gyrA*. (B) Capsular HA production was quantified at ML. (C) Lancefield assay for quantification of GAS survival in whole human blood. (D–F) Analysis of the impact of single gene replacement of *rocA*_M18_ with *rocA*_M89_ in hyper-encapsulated GAS-M18. (D) Absolute copy number of *hasA* transcripts quantified at EL growth phase relative to housekeeping gene *gyrA*. (E) Capsular HA production was quantified at ML growth phase. (F) Lancefield Assay for quantification of GAS survival in whole human blood. Data represent mean and standard deviation for three independent experiments measured in triplicate (Unpaired t-test;* = p<0.05, ** = p<0.01, *** = p<0.001).

The role of RocA in GAS virulence was further characterized by the creation of two allelic exchange mutants using GAS-M89 and GAS-M18, whereby the *rocA* gene of one was replaced with the *rocA* gene of the other, generating strains GAS-M89_rocAM18_ and GAS-M18_rocAM89_ respectively (Figure 4) (Table 2). The serotype M18 RocA truncation was firstly demonstrated to be sufficient to enhance encapsulation in a poorly encapsulated strain GAS-M89 (Figure 5 A and B). Loss of functional RocA in GAS-M89 resulted in a 3.5-fold increase in levels of *hasA* transcription (Figure 5 A) and an increase in detectable capsular HA synthesis from undetectable to 30 fg/cfu (Figure 5 B). This encapsulation phenotype was associated with enhanced GAS survival in the presence of whole human blood with 3.5-fold greater growth compared with wildtype GAS-M89 in a standard Lancefield assay (Figure 5 C).

Conversely, serotype M18 hyper-encapsulation was reversed by expression of a single chromosomal copy of full-length *rocA_M89_* in strain GAS-M18. *hasA* transcript levels were significantly reduced compared with GAS-M18 (Figure 5 D) and resulted in a dramatic reduction in capsular HA associated with GAS-M18_rocAM89_ (Figure 5 E). Survival of GAS-M18_rocAM89_ in whole human blood was significantly impaired compared with GAS-M18, with an 80% reduction in growth in this environment, attributable to the reversal of the highly encapsulated phenotype (Figure 5 F).

The cysteine protease SpeB is negatively regulated by CovR, though CovS opposes this effect [30]. SpeB expression is therefore abrogated in GAS strains where CovS-mediated regulation is impaired either as a result of CovR or CovS mutations. As such, SpeB expression has been used as a marker of CovR/S functionality [30]–[32]. To determine whether the non-synonymous CovR mutations identified in serotype M18 GAS were phenotypically silent (with respect to CovS function), we measured SpeB expression in all isogenic strains used in this study. SpeB was expressed by GAS-M18, and, furthermore, over-expression of CovR_M89_ in GAS-M18 did not impact on this (Figure S1). Whilst an increase in SpeB abundance was observed in strain GAS-M18_rocAM89_, the expression levels were comparable with capsule disruption mutant GAS-M18_hasKO_, suggesting the difference was an artifact due to reduction in capsule expression compared with GAS-M18, rather than a regulatory effect.

### The RocA mediated regulation of *covR/S*

**Figure 6:**
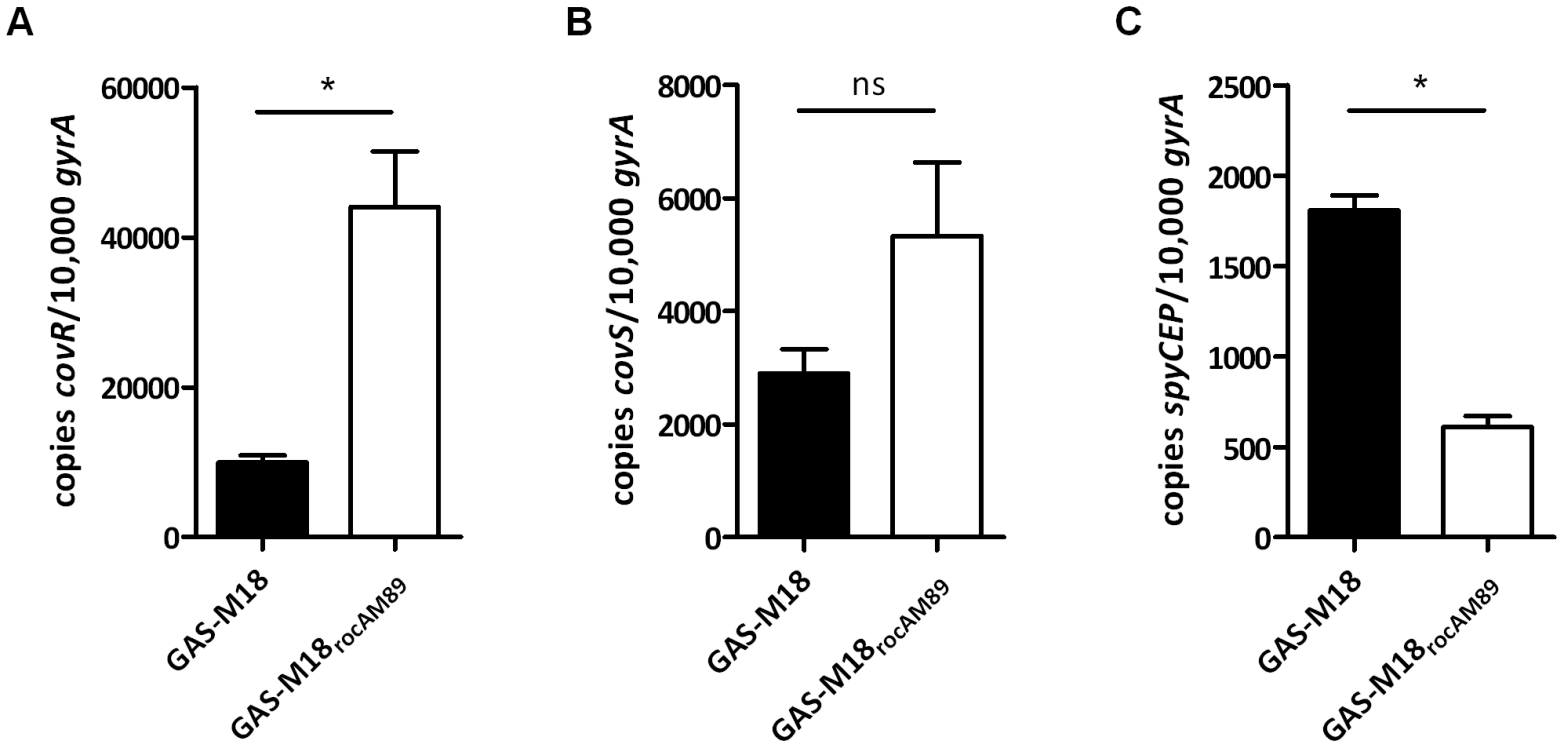
Functional RocA enhances *covR* but not *covS* transcript levels. RT-PCR analysis of isogenic strains GAS-M18 and GAS-M18_rocAM89_ to ascertain the impact of RocA on expression of global regulator CovR. Quantification of absolute copy number of (A) *covR*, (B) *covS* and (C) *spyCEP* transcripts relative to housekeeping gene *gyrA* at EL growth phase. Data represent mean and standard deviation for three independent experiments measured in triplicate (un-paired t-test, * = p<0.05).

RocA was reported previously to positively regulate transcription of *covR* [28], providing an explanation for the observed negative impact on *hasA*, which is regulated by CovR/S. Introduction of a single copy of functional *rocA_M89_* to GAS-M18 resulted in a marked 4-fold increase in *covR* transcription (Figure 6 A) but the impact on *covS* transcript levels was not significant (Figure 6 B). Consistent with increased abundance of CovR, transcription of *spyCEP*, a CovR-repressed virulence factor, was found to be significantly reduced in GAS-M18_rocAM89_ (Figure 6 C). Taken together these data demonstrate that functional RocA positively regulates the transcription of *covR*, with a downstream repressive effect on the CovR transcriptome. Replacement of *rocA*_M18_ with *rocA*_M89_ had no effect on *in vitro* superantigen production by M18 GAS (data not shown), demonstrating the specificity of the RocA regulatory network.

### The RocA regulon extends beyond the HA capsule and CovR/S

**Figure 7:**
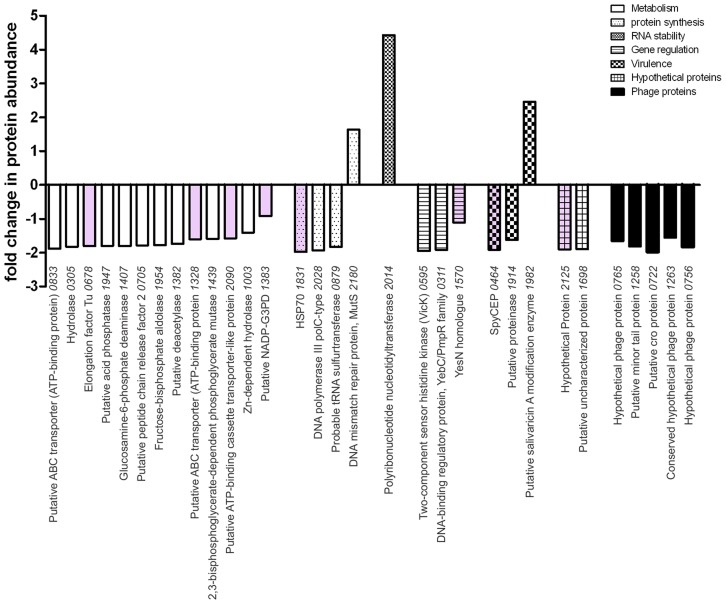
RocA has targeted impact on GAS proteome that is distinct from the CovR/S regulon. The impact of expressing full-length RocA_M89_ on the GAS-M18 proteome was ascertained by quantitative SDS-PAGE LC-MS/MS using cell pellets obtained from 4 independent cultures of isogenic strains GAS-M18 and GAS-M18_rocAM89_. Values represent relative protein abundance (fold-change) in strain GAS-M18_rocAM89_ relative to parent strain GAS-M18. Pink shading highlights genes included in the CovR/S regulon. Only proteins with altered expression of >1.5 fold and p≤0.05 were included. Numbers on x-axis represent M18 strain MGAS8232 ORFs.

*In silico* analysis of the RocA amino acid sequence demonstrated clear structural homology with the catalytic domain of a large number of sensor histidine kinases, notably the *Escherichia coli* osmoregulator EnvZ (http://www.sbg.bio.ic.ac.uk/~mwass/combfunc/). To determine the regulatory remit of RocA, quantitative mass spectrometry analysis was carried out on bacterial cell pellets obtained from GAS-M18 and GAS-M18_rocAM89_ grown to mid-logarithmic growth phase in THB. 1259 GAS proteins were identified in total, representing 69% of the serotype M18 proteome [7]. Of these proteins, 2.5% (31/1259) were differentially expressed, the majority of which were down-regulated in GAS-M18_rocAM89_ compared with wildtype GAS-M18 (28/31), while three were up-regulated (Table S1) (Figure 7). Intriguingly, of the 28 proteins down-regulated by RocA, half were identified to be involved in bacterial metabolism, which may well impact on capsule synthesis in serotype M18 GAS. Indeed two of these proteins are involved in synthesis of the HA precursor N-acetylglucosamine, glucosamine-6-phosphate deaminase and peptidoglycan N-acetylglucosamine deacetylase (ORF 1407 and 1382, Table S1). Of the 31 proteins differentially expressed only eight (Figure 7, pink shading; Table S1, bold font) are reported to be regulated by CovR/S [13], suggesting that the RocA regulon is complementary to but distinct from the CovR/S regulon. Furthermore, RocA was shown to control expression of at least two additional two component regulators as well as a regulator of RNA stability [33], underlining the complexity of GAS virulence regulation networks.

### RocA activity and capsule impact on GAS colony structure

**Figure 8:**
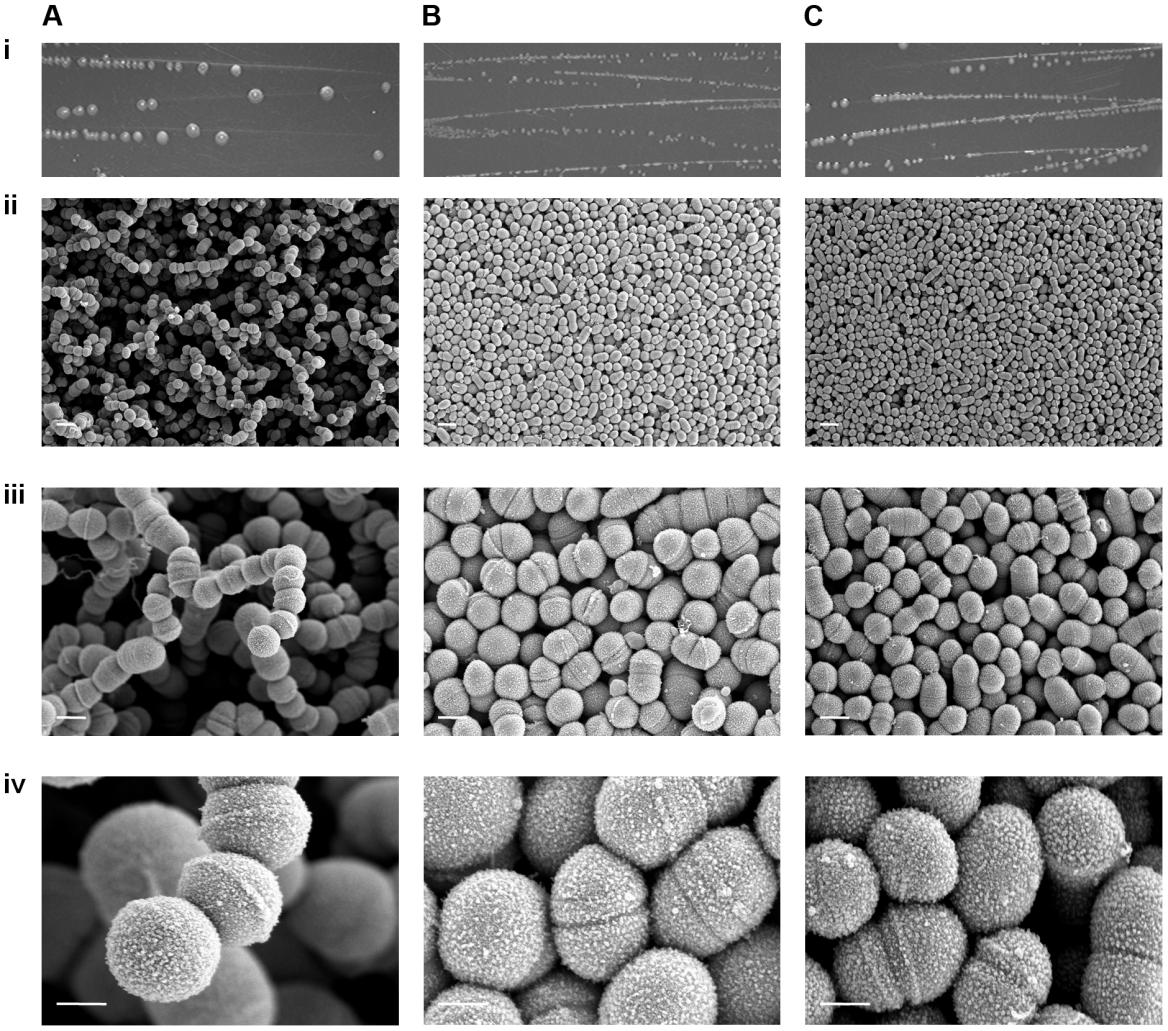
RocA regulation of capsule synthesis modulates bacterial colony structure. Imaging of serotype M18 strains (A) GAS-M18, (B) GAS-M18_rocAM89_ and (C) GAS-M18_hasko_ following overnight culture on Todd-Hewitt agar. i) Macroscopic imaging. ii)–iv)Scanning EM performed on individual colonies, each one imaged at three magnifications: ii) 3 k (white line = 2 µm), iii) 10 k (white line = 1 µm) and iv) 35 k (white line = 0.5 µm) respectively.

Scanning EM was undertaken on strains GAS-M18, GAS-M18_hasko_ and GAS-M18_rocAM89_ to ascertain the impact of hyper-encapsulation on the structural morphology of the resulting bacterial colonies (Figure 8 A–C). Due to the fragile nature of the association between capsular HA and GAS cocci it was not possible to preserve the capsule during the fixing process, however the structure of individual bacterial colonies was maintained. Scanning EM of colonies demonstrated that M18 hyper-encapsulation was associated with a unique morphology in 3D colony structure whereby chains of cocci protruded perpendicular to the colony plane (Figure 8 A). This was in stark contrast to the flat morphology exhibited by GAS-M18_rocAM89_ (Figure 8 B) and acapsular GAS-M18_hasko_ (Figure 8 C). This structural phenotype may be induced by charge repulsion between adjacent HA polymers or the accumulation of HA between GAS cocci, and may play a role in serotype M18 associated disease aetiology.

### Hyper-encapsulation induced by RocA truncation underlies serotype M18 carriage longevity in the murine nasopharynx and transmissibility

**Figure 9:**
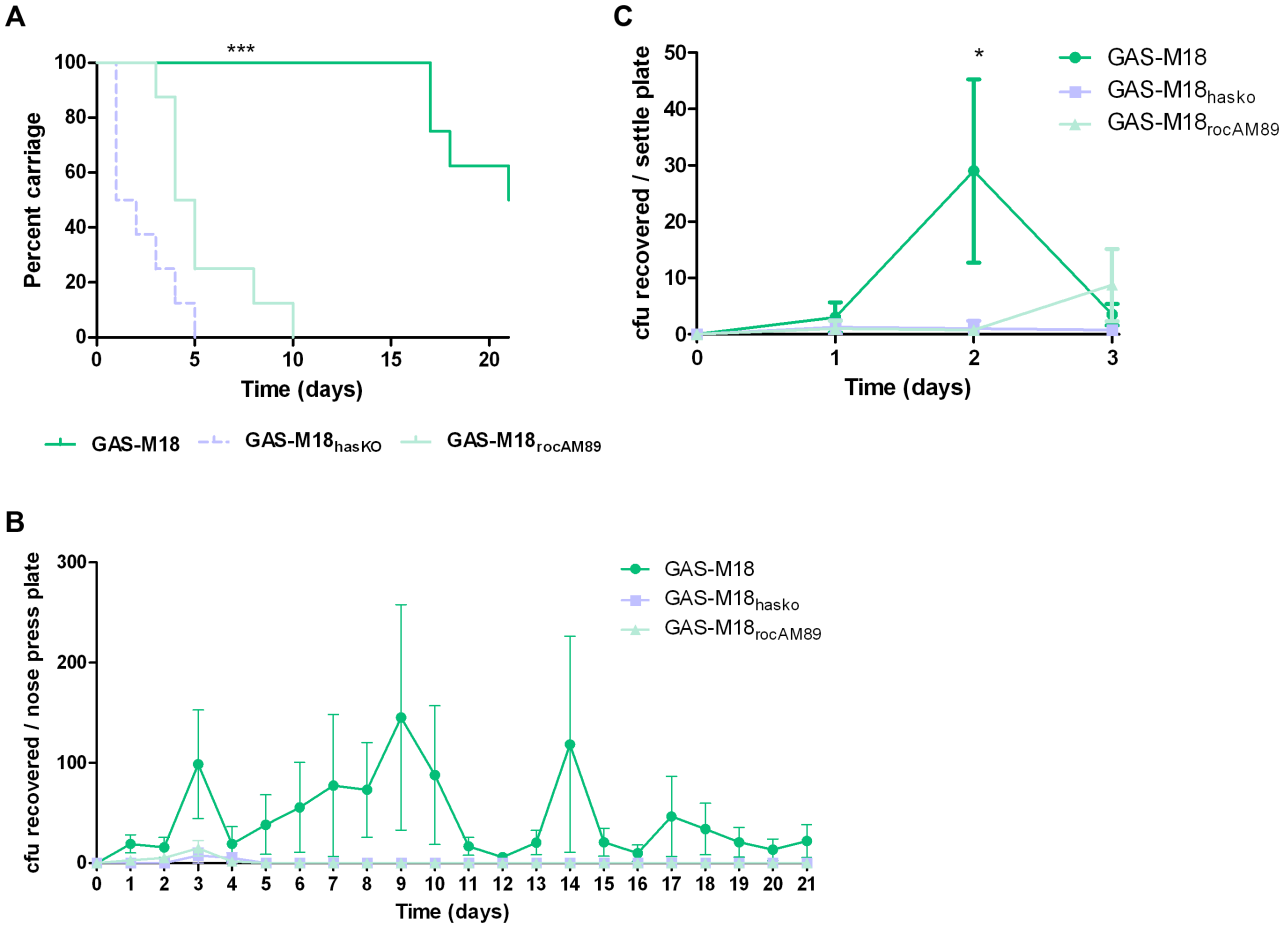
GAS-M18 nasopharyngeal carriage longevity and airborne spread require hyper-encapsulation induced by RocA truncation. (A) Quantification of murine nasopharyngeal carriage longevity of isogenic GAS strains GAS-M18, GAS-M18_hasKO_ and GAS-M18_rocAM89_ by nose-pressing (n = 8/group). Data represent percentage of mice colonized with each strain for 21 days following intra-nasal challenge (LogRank; *** = p<0.001). (B) Quantification of bacterial shedding as mean number of GAS cfu recovered from the mouse nasopharynx by daily nose-pressing onto individual blood agar plates (AUC and Kruskal Wallis (p<0.05)). (C) Quantification of airborne transmission of GAS to settle plates during the first 3 days of infection (n = 4 plates/cage) (AUC and Kruskal-Wallis; * = p<0.05).

Correction of the serotype M18 RocA truncation and subsequent reversal of the hyper-encapsulation phenotype led to a clear reduction in GAS carriage longevity in mice, whereby GAS-M18 persisted for significantly longer in the murine nasopharynx than GAS-M18_rocAM89_ following intra-nasal infection (Figure 9 A and B). Indeed, nasopharyngeal carriage longevity of GAS-M18_rocAM89_ was comparable to that of the isogenic acapsular *hasA* disruption mutant, GAS-M18_hasKO_, suggesting that the impact of RocA on capsule synthesis was a key determinant in GAS carriage longevity, rather than other effects of the RocA regulon. The disparity in nasopharyngeal carriage longevity was associated with enhanced airborne GAS transmission to blood agar settle plates placed above cages for the first three days following infection (Figure 9 C) even though nasopharyngeal GAS carriage was equivalent between groups at this time point. Enhanced carriage longevity, transmissibility and shedding of hyper-encapsulated GAS-M18 may go some way to explain the strong association between this serotype and outbreaks of pharyngitis and ARF.

## Discussion

The hyper-encapsulation of serotype M18 GAS has long been documented, but the underlying mechanism for this phenotypic phenomenon has remained elusive. In this investigation we have identified the cause of mucoidy in our collection of M18 strains as a naturally occurring truncation in the regulatory protein RocA, unique to, and conserved within the serotype M18 GAS isolates studied. This truncation is both necessary and sufficient to induce serotype M18 hyper-encapsulation.

Unique mutations were identified in the M18 GAS coding sequences of both the response regulator *covR* and in *rocA*. To determine the major influence on M18 hyper-encapsulation, full-length RocA_M89_ and CovR_M89_ were over-expressed in GAS-M18. Only full-length RocA_M89_ was sufficient to restore both transcriptional repression of the *has* operon and downstream reduction in capsular HA, providing clear evidence of the involvement of RocA in the regulation of capsule synthesis. Over-expression of wildtype CovR_M89_ in GAS-M18 did not reduce serotype M18 hyper-encapsulation to the same extent as over-expression of full-length RocA_M89_, despite both regulators having comparable effects on *hasA* transcription.

The influence of RocA was further characterized through *rocA* allelic exchange mutagenesis. Expression of a single chromosomal copy of full-length *rocA*_M89_ reversed hyper-encapsulation in GAS-M18 in association with reduced transcription from the *has* operon. The introduction of full length RocA also increased expression of the two component control of virulence regulator, *covR*, consistent with an earlier report [28]. CovR is known to be involved in transcriptional regulation of over 100 genes, including *hasA* and *spyCEP*, a chemokine cleaving protease [24], [27], [34], [35]. Similar to *hasA*, transcription of *spyCEP* was also reduced in strain GAS-M18_rocAM89_, lending support to the existing evidence that RocA regulates the expression of several genes, in part via CovR/S [28].

**Figure 10:**
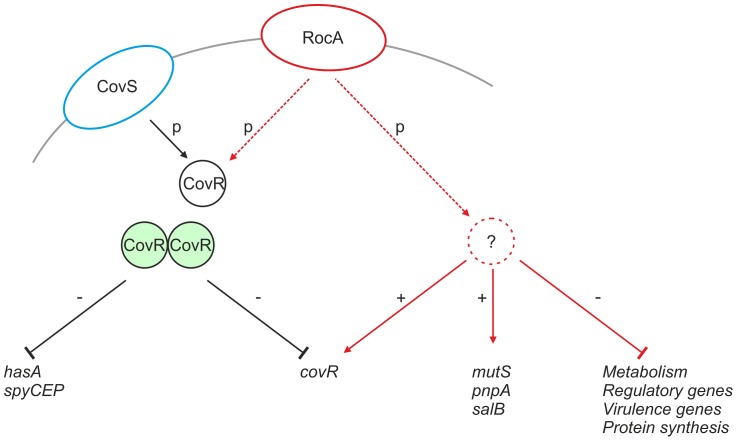
Proposed mechanism of RocA mediated activity. CovR (black circle) is phosphorylated directly by CovS (blue ellipse), which induces CovR dimerization (green circles) and transcriptional repression of *hasA* and *spyCEP*, as well as auto-repression of *covR*. RocA (red ellipse) regulates expression of a number of gene products (solid red lines). RocA promotes transcription of *covR* potentially via an intermediate regulator (red dashed circle). RocA also promotes expression of proteins that are not part of the CovR regulon, whilst inhibiting many others. Such regulation may also require kinase action and an intermediate regulator.

RocA shares structural homology with TCS sensor kinases, however is not located near to a cognate repressor protein in the GAS genome [28]. It is possible that RocA modulates expression of target genes, including *covR*, by functioning as a trans-acting kinase on one or more currently unidentified regulators that may include CovR (Figure 10). Although functional RocA positively enhanced *covR* transcription, baseline *covR* transcript levels were similar between M18 GAS and other serotypes tested, notwithstanding the M18 RocA truncation. We hypothesize that, while loss of RocA activity in M18 GAS would tend to reduce *covR* transcription, levels may be restored by consequent reduction in auto-repression of *covR* transcription, resulting from both a reduction in CovR protein levels and, potentially, a reduction in phosphorylated CovR because of reduced kinase activity.

Tight transcriptional regulation of virulence factors is critical to both survival and infection potential of GAS [17]–[23]. The loss of functional RocA in serotype M18 has the potential to impact on at least 10% of the GAS genome through a regulatory effect on the CovR/S TCS [24]. Our data suggest that, in the serotype M18 background, capsule production was most strongly affected by the RocA truncation. Indeed, the impact of the RocA truncation on *hasA* and capsule expression was an order of magnitude greater in the M18 strain background than in the M89 background. Although inter-serotype differences in gene regulation are not unprecedented [36], we considered the possibility that factors additional to the RocA truncation contributed to the GAS M18 hyper-encapsulation phenotype, specifically, the observed non-synonymous mutations in CovR_M18_. Binding of CovR_M18_ to the *has* promoter *in vitro* was not, however, impeded by the mutations we report (not shown), suggesting that the mutations do not impact on DNA binding. We cannot exclude the possibility that the mutations in CovR affect phosphorylation and coupling to CovS-mediated regulation however we found no evidence of SpeB repression in M18 GAS, further suggesting that the CovR mutations are likely to be silent [30], [32].

RocA dependent regulation in GAS was further elucidated by quantitative proteomic comparison of M18 GAS cell pellets with cells from an isogenic mutant expressing full-length RocA_M89_. 31 proteins out of 1259 identified by LC-MS/MS were differentially expressed between the two strains, nearly half of which were involved in metabolic processes, all demonstrating reduced expression in the presence of functional RocA protein. The regulation of genes connected to metabolism, protein synthesis, regulation and virulence demonstrates that RocA activity extends beyond a straightforward interaction with CovR/S (Figure 10). Intriguingly, despite detection of almost 70% of the entire M18 GAS proteome, analysis did not detect as many differentially expressed proteins as might be predicted by the reported CovR/S regulon [24]. Indeed only a quarter of proteins differentially regulated by RocA belonged to the known CovR/S regulon. In part this may be because RocA does not wholly control CovR/S transcription. It should be noted that bacteria were grown to early-mid logarithmic growth phase for the proteomic studies. Whilst optimal capsule production occurs at this time point, the expression pattern of other proteins differs considerably, and several CovR/S-regulated genes are influenced at late logarithmic or even stationary phases of growth [24]. Importantly we elected to measure differential protein expression rather than mRNA transcript abundance, as has been undertaken previously, in order to better identify the major candidate proteins likely to play a role in the M18 GAS phenotype. It is therefore not surprising that the number of differentially expressed proteins is less than has been found in transcriptomic studies.

Of note, a homologue to a bacterial metabolism regulator, YesN [37], was down-regulated in the presence of full-length functional RocA. This lends support to the hypothesis that RocA plays a direct role in the regulation of GAS metabolism. HA synthesis is a highly metabolic process, with both polysaccharide precursors stemming from glucose-6-phosphate [15]. Whilst the synthesis of glucuronic acid is catalyzed by the enzymes of the *has* operon, N-acetylglucosamine is a metabolite of cell wall biosynthesis. Our proteomic data, coupled with HA assays, suggest that the RocA truncation leads not only to increased *hasA* transcription, but also to increased expression of several components of the biosynthetic pathway that modulate the abundance of N-acetylglucosamine.

In some contrast to findings in the murine model reported herein, M18 GAS are infrequently found as causes of pharyngitis, although geographically defined outbreaks have been reported both from the mid-1980s and contemporary times, notably in association with the onset of ARF [6]–[10]. The impact of changes in population immunity and environment on M18 GAS epidemiology is unknown. Whether the propensity of M18 GAS to prolong nasopharyngeal infection can explain the association between M18 GAS and ARF is unclear. It has been hypothesized that the HA capsule per se may provide a basis for collagen-based autoimmunity through aggregation of collagen [38], however other studies point to a immunoregulatory role for HA on dendritic cell activation [39]. Importantly, lower molecular weight moieties of HA can act as immunostimulatory molecules acting via TLR4 [40]. As it is not known how GAS HA is processed or trafficked during human infection, the role of HA capsule in ARF remains uncertain.

The truncation of RocA in M18 GAS adds to the list of regulatory gene mutations reported to impact on GAS virulence, examples of which include not only mutations in *covR/S* that affect pleiotropic virulence factors including capsule, but also those in *rgg*/*ropB*, and *mtsR* [41], [42] which affect SpeB for example. Whilst mutations in many regulators arise spontaneously [24], [31], [32], often as a result of blood passage or exposure to host tissues, the *rocA*_M18_ mutation is unusual in that it was conserved among all 12 serotype M18 strains tested in this study, spanning an almost 80 year interval. Although further testing of many more isolates is required, we speculate that the mutation may be serotype defining.

In apparent contrast to *covR/S* mutations, which are associated with a fitness burden during upper respiratory tract infection [43] and adversely impact on biofilm formation [32], the RocA truncation conferred an ability to survive and transmit during colonization. While capsule may impede binding and biofilm formation by some GAS serotypes, the impact of capsule on survival in air, or at the surface of a pre-existing biofilm is unknown. In the case of M18 GAS, excess production of HA capsule appears essential to pathogenesis, perhaps by mediating binding to host proteins such as CD44 [4], [5] and does not appear to negatively impact on experimental pharyngeal infection.

Intriguingly, airborne transmission of GAS to blood agar plates placed within the cages was also dependent on the RocA truncation. This was not simply a consequence of differential carriage or shedding levels, since mice at this time point showed no difference in direct nasal transmission to blood agar or in bacterial counts in colonized nasal tissue, though may relate to survival on air-exposed surfaces (Lynskey unpublished). Bacterial and host factors that influence GAS airborne transmission are uncharacterized. Taken together, the data highlight the possibility that excessive capsular HA may augment persistence and transmission of GAS-M18 as a consequence of a conserved mutation in a metabolic regulatory gene.

## Materials and Methods

### Ethics statement

The use of anonymized human blood was approved through the Imperial College NHS Trust Tissue Bank. *In vivo* experiments were performed in accordance with the Animals (Scientific Procedures) Act 1986, and were approved by the Imperial College Ethical Review Process (ERP) panel and the UK Home Office.

### Bacterial strains and growth conditions

GAS isolates were collected from patients at ICHNT, or by the UK reference laboratory. The strains used for molecular manipulation were invasive disease isolates GAS-M18 (H566) and GAS-M89 (H293). GAS were cultured on Columbia horse blood agar plates (OXOID) Todd-Hewitt (TH) agar or in TH broth (OXOID) at 37°C, 5% CO_2_ for 16 hours. *E. coli* XL-10 gold (Stratagene) and DH5α (Invitrogen) were grown in LB broth. Growth media were supplemented with antibiotics where appropriate at the following concentrations; *E. coli* spectinomycin 50 µg/ml, kanamycin 50 µg/ml; GAS spectinomycin 50 µg/ml, kanamycin 400 µg/ml.

### Polymerase chain reaction and DNA sequencing

Genomic DNA was extracted from GAS cultures grown to late logarithmic growth phase (OD_600_ 0.7–0.9) as described previously [44]. PCR was carried out using a MyCycler (Bio-Rad) thermal cycler with Bio-X-Act proof reading Taq (Bioline). Automated-fluorescent sequencing of products was performed by the MRC CSC Core genomics laboratory, Hammersmith Hospital. For sequencing of *rocA* genomic DNA was amplified and sequenced using the following primers: forward primer 1: 5′- TTGCAAAAACTGTAGGCTGTG-3′ reverse primer 1: 5′- GCCAGGTTGAAAAATCGAAA-3′; forward primer 2: 5′-GCCATTGTTTGGTATGCCTTA-3′, reverse primer 2: 5′-GGGATCGATACCTCAACCTT-3′; forward primer 3: 5′- TGAAGGTATCTTGAATGCTGAAA-3′, reverse primer 3: 5′-GCTGAAATTTTAACTCTAGCTTGGA-3′.

### Construction of over-expression strains

For CovR_M89_ over-expression, the *covR* coding sequence, including native promoter, was amplified from GAS-M89 (forward primer: 5′- CGGGATCCACTGAATATTAAAGAGTGTCTGAA-3′, reverse primer: 5′- CGGGATCCTTGAACTATATGGCAATCAGTG-3′) incorporating BamHI restriction sites to both ends of the PCR product, and cloned into BamHI digested shuttle vector pDL278 [45] resulting in plasmid pDL_covRM89_.

For RocA_M89_ over-expression, the *roc*A coding sequence, including native promoter, was amplified from GAS-M89 (forward primer: 5′- GACGGATCCAATTCTTGCAAAAACTGTAGGCTGTC, reverse primer: 5′- GACGGATCCAATTCGCTGAAATTTTAACTAGCTTGGA-3′) incorporating BamHI restriction sites to both ends of the PCR product, and cloned into BamHI digested shuttle vector pDL278 [45], resulting in plasmid pDL_rocAM89_. For RocA_M18_ over-expression, the serotype M18 SNP (T to A at nucleotide 269) was incorporated into the pDL_rocAM89_ plasmid sequence by site-directed mutagenesis (QuikChange XL-II Site-Directed Mutagenesis Kit, Stratagene) (forward primer: 5′- CTATGGTAAATCAATAAAAGCTAAGTTTTAAATGTTTTATGCCTTTTTCCACTAGTG-3′, reverse primer: 5′- CACTAGTGGAAAAAAGGCATAAAACATTTAAAACTTAGCTTTTATTGATTTACCATAG-3′) to produce vector pDL_rocAM18_.

The sequence of *covR* or *rocA* in all vectors was confirmed by Sanger sequencing and the resulting plasmids, as well as empty pDL278 control vector, were introduced into GAS-M18 by electroporation. The successful introduction of plasmid was confirmed by PCR specific for pDL278 backbone (forward primer: 5′- CATTCAGGCTGCGCAACTG-3′, reverse primer: 5′- TCGAATTCACTGGCCGTCG-3′) in each of the resulting isogenic strains GAS-M18_pcontrol_ (containing empty vector), GAS-M18_pcovRM89_ (over-expressing CovR_M89_), GAS-M18_procAM89_ (over-expressing full-length RocA_M89_) and GAS-M18_procA-M18_ (over-expressing truncated RocA_M18_).

### Construction of *rocA* allelic exchange mutants

Full-length *rocA*_M89_ including native promoter was amplified from GAS-M89 (forward primer: 5′- GACGGATCCAATTCTTGCAAAAACTGTAGGCTGTC, reverse primer: 5′- GACGGATCCAATTCGCTGAAATTTTAACTAGCTTGGA-3′), incorporating EcoRI restriction sites to both ends of the PCR product, and cloned into EcoRI digested suicide vector pUCMUT [46]. The resulting plasmid, pUCMUT_rocAM89_, was confirmed to encode full-length *rocA* by Sanger sequencing. In order to create an allelic exchange vector to introduce *rocA*_M18_ into GAS of a different serotype, the serotype M18 SNP (T to A at nucleotide 269) was incorporated into the pUCMUT_rocAM89_ plasmid sequence by site-directed mutagenesis (QuikChange XL-II Site-Directed Mutagenesis Kit Stratagene) (forward primer: 5′- GCTGAAAAGAATAATGCTAAAGATGACAGACTTGATTTAACTTGTTTAGATAAAT-3′, reverse primer: 5′- ATTTATCTAAACAAGTTAAATCAAGTCTGTCATCTTTAGCATTATTCTTTTCAGC-3′). The change in *rocA* sequence was confirmed by Sanger sequencing and DraI digest. To introduce a second region of homology with the bacterial chromosome, approximately 500 bp of downstream conserved sequence including the 3′ region of *spy_1611* was amplified (forward primer: 5′-CGCCGTCGACTTATTGTTTCTTCCAAGCTAG, reverse primer: 5′-CGCCTGCAGGGAGTCACTATTGGTACTAT-3′) incorporating PstI and SalI restriction sites to the 5′ and 3′ of the PCR product respectively, and cloned into PstI/SalI digested pUCMUT_rocAM89_ and pUCMUT_rocAM18_, to produce allelic exchange vectors pUCMUT_rocAM89_AE and pUCMUT_rocAM18_AE respectively. pUCMUT_rocAM89_AE was introduced into GAS-M18 by electroporation and crossed into the chromosome by homologous recombination (Figure 4). Double allelic exchange was confirmed by PCR and Sanger sequencing of the *rocA* gene. Polar effects were not expected due to the orientation of the genes surrounding *rocA*, and were ruled out by quantitative proteomic analysis of flanking gene products. pUCMUT_rocAM18_AE was introduced into GAS-M89 by electroporation as detailed for pUCMUT_rocAM89_AE and GAS-M18.

### Construction of a *hasA* disruption mutant

A 500 bp fragment of the 5′ *hasA* gene was amplified (forward primer: 5′- GGGGTACCTATCTTGATTTATCTAAATATG-3′, reverse primer: 5′- GGAATTCGTTTCTAGCATTCAAATGTCCT-3′) incorporating EcoRI and KpnI restriction sites into the 5′ and 3′ ends respectively, and cloned into the suicide vector pUCMUT to produce vector pUCMUT_hasA_. A 500 bp fragment of the 3′ *hasB* gene was amplified (forward primer: 5′- ACGCGTCGACATGATGATCGAATAGGAATGC-3′, reverse primer: 5′- AACTGCAGCAATCATACCACCAACTGCAG-3′) incorporating PstI and SalI restriction sites into the 5′ and 3′ ends respectively, and cloned into PstI/SalI digested pUCMUT_hasA_. The construct was introduced into GAS-M18 by electroporation and crossed into the chromosome by homologous recombination. PCR analysis demonstrated that only a single recombination event between chromosomal *hasA* and the 500 bp 5′ *hasA* fragment had occurred. The insertion was stable following murine intra-nasal infection, and was sufficient to disrupt capsule biosynthesis, demonstrated by quantification of capsular HA.

### Quantitative real-time PCR

RNA was extracted from GAS at early, mid and late logarithmic growth phases and converted to cDNA following DNase treatment with TurboDNAfree (Ambion, Cambridgeshire UK) as described previously [27]. qrtRT-PCR was carried out for the genes *hasA*, *covR* and *covS*, and expression data normalized to that of *gyrA* using a standard curve method as described previously [27].

### Human whole blood phagocytosis assay

Lancefield assays were performed to assess GAS resistance to human phagocytic killing. GAS were cultured to OD_600_ 0.15 in THB, and diluted in sterile PBS. Approximately 50 GAS cfu were inoculated into heparinized whole human blood obtained from healthy volunteers, and incubated for 3 hours at 37°C with end-over-end rotation. Bacterial survival was quantified as multiplication factor of number of surviving colonies relative to the starting inoculum. Each strain was cultured in blood from three donors and tested in triplicate.

### Measurement of cell-associated hyaluronic acid

GAS capsular HA was extracted as described previously [6]. Briefly, GAS were cultured to mid logarithmic growth phase and washed twice in 10 mM Tris (pH 7.5). Capsule was removed following incubation with an equal volume of chloroform for 30 minutes with vortexing followed by a 60 minute static incubation at room temperature. Quantification of eluted capsular HA was carried out using the hyaluronan DuoSet ELISA (R&D). Data were standardized for total GAS cfu which were calculated in triplicate for each sample.

### Murine intra-nasal infection

FVB/n female mice (4–5 weeks old (Charles River, Margate,UK)) were briefly anaesthetized with isofluorane and challenged intra-nasally with 1×10^7^ GAS cfu, administered as 5 µl per nostril.

### Quantification of nasopharyngeal carriage longevity

Nasal carriage was longitudinally and non-invasively monitored daily for 21 days following intra-nasal challenge using a nose-pressing technique [43]. Briefly, mice were scruffed and their noses pressed gently into a CBA plate (Oxoid) 10 times. Resulting exhaled moisture was spread over the plate and colonies counted following incubation at 37°C, 5% CO_2_ for 24 hours. On day 21 mice were euthanized, and nose, cervical lymph node, spleen and lung dissected and plated to determine nasal colonization and systemic dissemination of GAS. Strains GAS-M18, GAS-M18_hasKO_, GAS-M18_rocAM89_, M4, M6 and M12 were compared over 21 days (Table 1). To detect airborne transmission of bacteria within cages of infected mice, CBA settle plates (4 per cage) were placed face up on the upper rack of individually HEPA filtered cages for 4 hours on days −1, 0, 1 and 2 post-infection as previously reported [43]. Airborne bacteria were quantified following overnight incubation of plates at 37°C, 5% CO_2_.

### Preparation of GAS samples for quantitative proteomic analysis

GAS cultures (8 ml) were grown on 4 separate occasions to OD_600_ 0.4 in THB supplemented with hyaluronidase (30 µg/ml) (Sigma). Bacterial pellets were washed twice and resuspended in 100 µl Tris-HCL (10 mM, pH 7.5) and stored in 100 mM DTT and 1× Lithium dodecyl sulphate (LDS) (Invitrogen) after heating at 75°C for 10 minutes. Samples were diluted according to protein concentration.

### Preparation of samples for 1D-gel-liquid chromatography mass spectrometry

Proteins were separated by 1-D electrophoresis and processed for Reverse Phase-nano-liquid chromatography mass spectrometry as described previously [47]. Data were analyzed using Progenesis software (Nonlinear Dynamics, USA). Data filters were set such that peptides included were single, double or triple charged and above threshold values for cross-correlation score. Differential protein production was deemed significant where a p value≤0.05 was obtained for a fold change ≥1.5. Results were validated by western blot for a selection of differentially expressed proteins using rabbit anti-sera raised against SpyCEP [48], NADP-G3PD, HSP70 and fructose bis-phosphate aldolase [47] (Figure S2).

### GAS protein preparation and western blot

GAS protein samples for validation of quantitative proteomic analysis were obtained as outlined above. Protein samples for quantification of SpeB expression were prepared from supernatants of GAS grown to stationary phase, which were 7× concentrated using 10 kDa cutoff spin columns (Amicon). For SDS-PAGE, samples were denatured with 100 mM DTT and 1× Lithium dodecyl sulphate (LDS) (Life technologies) and heated at 75°C for 10 minutes. Proteins were fractionated on either 10% NuPAGE novex bis-tris gels or 7% NuPAGE Tris-acetate gels (Life Technologies) for optimal separation of proteins within the desired molecular weight range. For western blot, proteins were transferred onto a Hybond-P membrane (Amersham) and blocked with blocking solution (5% milk (Sigma) with 0.05% Tween-20 (Sigma)). Blots were probed with either anti-SpeB antibody (Toxin Technology), anti-SpyCEP rabbit antiserum [48] or rabbit antiserum raised to the unique c-terminal pentapeptide of proteins fructose bisphosphate aldolase, NADP-G3DP or HSP70 [47] diluted 1∶1000 in blocking solution overnight at 4°C. Proteins were detected following incubation with HRP-conjugated anti-rabbit secondary (Life Technologies) diluted 1∶80,000. Membrane development was carried out with ECL Advance western blotting detection kit (GE Healthcare).

### Scanning electron microscopy

Pieces of agar 5 mm^2^ surrounding individual colonies were cut directly from petri dishes and fixed in 2.5% glutaraldehyde and 4% PFA in 0.01 M PBS for 1 hour, rinsed in 0.1 M sodium cacodylate buffer for 3×5 minutes and fixed again in 1% buffered osmium tetroxide for 1 hour. For better conductivity the samples were further impregnated with 1% aqueous thiocarbohydrazide and osmium tetroxide layers separated by sodium cacodylate washes following the protocol for OTOTO [49]. The colonies were then dehydrated in an ethanol series 30%, 50%, 70%, 90% and 100% (×3) for 20 minutes each and critical point dried in a Bal-Tec CPD030 before mounting onto aluminium stubs with silver dag and sputter coating with a 2 nm gold layer in a Bal-Tec SCD050. Examination and imaging was performed on an Hitachi S-4800 scanning electron microscope.

### Statistical analysis

All statistical analyses were performed with GraphPad Prism 5.0. Comparison of two datasets was carried out using unpaired students t-test and three or more data sets were analyzed by Kruskal-Wallis followed by Dunn's multiple comparison test or ANOVA and Bonferroni post-test depending on sample size. Survival data were analyzed by Mantel-Cox (Log rank) test. A p-value of ≤0.05 was considered significant.

## Supporting Information

Figure S1 SpeB expression in isogenic derivatives of GAS-M18. Bacterial supernatants obtained from stationary phase cultures of parent strain GAS-M18 and isogenic strains used in this study were fractionated by SDS-PAGE and immunoblotted with polyclonal rabbit anti-SpeB antibody. SpeB production was detected in the parent strain despite CovR mutations. Differences in SpeB abundance were observed only as a result of changes in capsule expression.
(TIF)

Figure S2 Validation of quantitative proteomics by western blot. Western blot analysis was carried out on cell pellets obtained from three independent cultures as outlined in the Methods section for four proteins detected in lower abundance in GAS-M18_rocAM89_ compared with GAS-M18 by quantitative LCMS/MS. Multiple bands for SpyCEP blot reflect the known products of autocatalytic cleavage. Relative abundance of each protein was estimated using ImageJ software. Fold-change in protein abundance averaged over the three experiments is shown compared with that calculated by LCMS/MS.
(TIF)

Table S1 Differential expression of proteins regulated by RocA. Data show fold-change of proteins differentially expressed >1.5-fold in GAS-M18 (n = 31) (Mann-Whitney p≤0.05). ORF numbers relate to genome sequenced M18 isolate MGAS8232 [7]; bold text indicates genes in the CovR/S regulon [13]. Grey shading indicates proteins with increased expression in GAS-M18_rocAM89_ (n = 3/31) ie: increased by functional RocA. ∧ Indicates proteins visualized by western blot for validation of the experiment.
(RTF)
