# Supplementary figures and images for: RocA Truncation Underpins Hyper-Encapsulation, Carriage Longevity and Transmissibility of Serotype M18 Group A Streptococci
Source: PLoS Pathog. 2013 Dec 19;9(12):e1003842. doi: 10.1371/journal.ppat.1003842 (PMC3868526; doi:10.1371/journal.ppat.1003842)

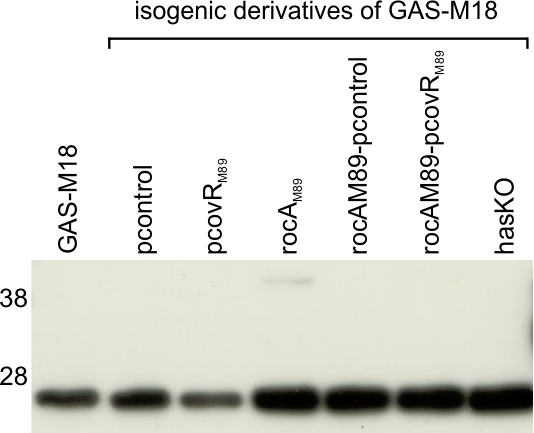

Supplement: Figure S1 — SpeB expression in isogenic derivatives of GAS-M18. Bacterial supernatants obtained from stationary phase cultures of parent strain GAS-M18 and isogenic strains used in this study were fractionated by SDS-PAGE and immunoblotted with polyclonal rabbit anti-SpeB antibody. SpeB production was detected in the parent strain despite CovR mutations. Differences in SpeB abundance were observed only as a result of changes in capsule expression. (TIF) [file ppat.1003842.s001.tif]

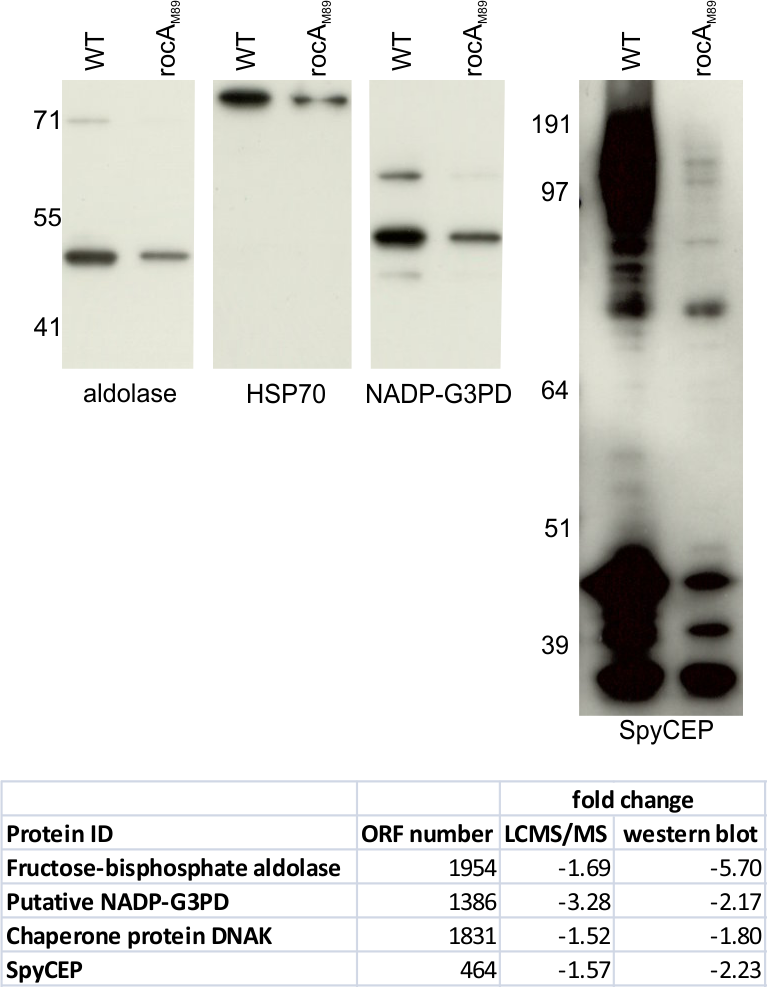

Supplement: Figure S2 — Validation of quantitative proteomics by western blot. Western blot analysis was carried out on cell pellets obtained from three independent cultures as outlined in the Methods section for four proteins detected in lower abundance in GAS-M18rocAM89 compared with GAS-M18 by quantitative LCMS/MS. Multiple bands for SpyCEP blot reflect the known products of autocatalytic cleavage. Relative abundance of each protein was estimated using ImageJ software. Fold-change in protein abundance averaged over the three experiments is shown compared with that calculated by LCMS/MS. (TIF) [file ppat.1003842.s002.tif]
