# Supplementary material for: RocA Truncation Underpins Hyper-Encapsulation, Carriage Longevity and Transmissibility of Serotype M18 Group A Streptococci
Source: PLoS Pathog. 2013 Dec 19;9(12):e1003842. doi: 10.1371/journal.ppat.1003842 (PMC3868526; doi:10.1371/journal.ppat.1003842)
Supplement: Table S1 — Differential expression of proteins regulated by RocA. Data show fold-change of proteins differentially expressed >1.5-fold in GAS-M18 (n = 31) (Mann-Whitney p≤0.05). ORF numbers relate to genome sequenced M18 isolate MGAS8232 [7]; bold text indicates genes in the CovR/S regulon [13]. Grey shading indicates proteins with increased expression in GAS-M18rocAM89 (n = 3/31) ie: increased by functional RocA. ∧ Indicates proteins visualized by western blot for validation of the experiment. (RTF) [file ppat.1003842.s003.rtf]

Protein identification	ORF number	fold change	
Metabolism:
Putative ABC transporter (ATP-binding protein)	
0833	
-1.59	
Hydrolase	0305	-1.65	
Elongation factor Tu	0678	-1.66	
Putative acid phosphatase 	1947	-1.67	
Glucosamine-6-phosphate deaminase	1407	-1.67	
Putative peptide chain release factor 2 	705	-1.68	
Fructose-bisphosphate aldolase^ 	1954	-1.69	
Peptidoglycan N-acetylglucosamine deacetylase	1382	-1.73	
Putative ABC transporter (ATP-binding protein)	1328	-1.87	
2,3-bisphosphoglycerate-dependent phosphoglycerate mutase	1439	-1.89	
Putative ATP-binding cassette transporter-like protein	2090	-1.90	
Zn-dependent hydrolase	1003	-2.14	
Putative NADP-dependent glyceraldehyde-3-phosphate dehydrogenase^	1383	-3.28	
Protein synthesis:
Chaperone protein DNAK OS^	
1831	
-1.52	
DNA polymerase III polC-type	2028	-1.55	
Probable tRNA sulfurtransferase 	0879	-1.64	
Gene regulation:
Two-component sensor histidine kinase (VicK)	
0595	
-1.54	
DNA-binding regulatory protein, YebC/PmpR family	0311	-1.57	
YesN homologue	1570	-2.71	
Virulence:
SpyCEP^	
0464	
-1.57	
Putative proteinase	1914	-1.85	
Hypothetical proteins:
Hypothetical Protein	
2125	
-1.57	
Putative uncharacterized protein 	1698	-1.58	
Phage proteins:
Hypothetical phage protein	
0765	
-1.82	
Putative minor tail protein	1258	--1.64	
Putative cro protein 	0722	-1.52	
Conserved hypothetical phage protein
Hypothetical phage protein	1263
0756	-1.56
-1.65	
DNA mismatch repair protein, MutS (protein synthesis)	2180	1.64	
Polyribonucleotide nucleotidyltransferase (RNA stability)	2014	4.43	
Putative salivaricin A modification enzyme (virulence)	1982	2.46	
